# Altered Tumor Plasticity after Different Cancer Cell Fusions with MSC

**DOI:** 10.3390/ijms21218347

**Published:** 2020-11-06

**Authors:** Catharina Melzer, Juliane von der Ohe, Ralf Hass

**Affiliations:** Biochemistry and Tumor Biology Lab, Department of Obstetrics and Gynecology, Hannover Medical School, 30625 Hannover, Germany; catharina.melzer@t-online.de (C.M.); Ohe.Juliane.von.der@mh-hannover.de (J.v.d.O.)

**Keywords:** cancer cell fusion, mesenchymal stroma/stem cells, tumor heterogeneity, aneuploidy, post-hybrid selection process

## Abstract

While cell fusion demonstrates an important pathway during tissue development and regeneration of distinct organs, this process can also contribute to pathophysiological phenotypes during tumor progression. Hybrid cell formation after heterofusion between cancer cells and various other cell types within the tumor microenvironment is observed in vitro and in vivo. In particular, mesenchymal stroma/stem-like cells (MSC) perform diverse levels of communication with cancer cells by exhibiting anti- and pro-tumorigenic effects. During these cellular interactions, MSC can eventually fuse with cancer cells. Thereby, the newly generated disparate hybrid populations display aneuploidy associated with chromosomal instability. Based upon a subsequent post-hybrid selection process (PHSP), fused cancer cells can undergo apoptosis/necroptosis, senescence, dormancy, or a proliferative state by acquisition of new properties. Consequently, PHSP-surviving hybrid cancer cells demonstrate altered functionalities within the tumor tissue. This is accompanied by changes in therapeutic responsiveness and a different metastatic behavior. Accordingly, enhanced tumor plasticity interferes with successful therapeutic interventions and aggravates patient prognoses. The present review article focusses on fusion of MSC with different human cancer cells, in particular breast cancer populations and resulting characteristics of various cancer hybrid cells. Moreover, some mechanisms of cancer cell fusion are discussed together with multiple PHSP pathways.

## 1. Introduction

Cell fusion represents a physiological process that is required during development of certain tissues. This includes the fusion of myoblasts to form multinucleated myocytes in muscle fibers during the development of muscle tissue. Fusion of fetal trophoblasts occurs to evolve syncytiotrophoblasts during the formation of placenta barrier and tissue [1,2]. These processes of homofusion as characterized by the fusion of cells from the same population are also termed autofusion. Conversely, heterotypic fusion or heterofusion describes hybrid formation of different cell types [3]. Fusion of different mononuclear precursor cells provides an example for heterofusion contributing to osteoclast formation for the maintenance, repair, and remodeling of bone tissue [4]. These normal development-associated fusion processes are tightly regulated. Alternatively, the formation of hybrid cells can also occur spontaneously by so called “accidental cell fusion”. This apparently unconstrained process is supported by transient establishment of a fusion-permissive environment, including acidic pH, hypoxia, accumulation of damage-associated molecular patterns, and membrane lipids destabilizing ions and peptides [5,6].

In addition to developmental properties, cell fusion is also involved in regenerative activities. Following transplantation of bone marrow cells, including bone marrow-derived mesenchymal stroma/stem-like cells (MSC) to appropriate tissues, cell fusion can be observed with skeletal muscle cells, cardiomyocytes, hepatocytes, and Purkinje cells [7]. While cell fusion of two somatic cells results in tetraploidy, physiological processes with regenerative requirements can take advantage of tetraploid cell populations with preferably mesenchymal origin. In particular, fusion processes can contribute to regeneration of liver tissue [8].

Conversely, cell fusion can also display the basis for pathophysiological developments such as cancer. Although fusion processes during neoplastic degeneration are considered rare events, their actual frequency may be much higher according to postulated “hidden” fusions [9]. Whereas cell fusion can generate aneuploidy, chromosomal instability, and DNA damage, these pathways cause multiple genetic aberrations and potentially new or altered neoplastic development [10].

Cancer cell fusion is observed with distinct cell types, including leukocyte-tumor cell fusions [11] or macrophage-tumor cell fusions. These include, e.g., lung cancer, gastric cancer, brain metastases of melanoma, different tumors of the breast, and bone marrow-derived cells [2,12,13,14,15,16]. Another predominant fusion partner in tumor tissue is represented by MSC [17,18,19].

## 2. MSC Functionality and Tumor Interactions

Important functionalities of MSC in adult human tissues include repair mechanisms and regenerative activities. MSC exhibit immune-modulatory capabilities, paracrine effects, and antimicrobial functions during various physiological processes. These multiple functionalities are based at least in part on the heterogeneity of MSC populations, although characteristics and the biological role of this MSC diversity remain only partially understood. Primary MSC can be derived from perivascular regions with distinct properties according to the various originating adult organs and tissues whereby superior in vitro growth potential and regenerative capacity are observed in MSC populations from neonatal materials such as placenta or umbilical cord [20]. According to this heterogeneity, MSC are characterized by a set of minimal criteria like in vitro plastic adherence, migratory activity [21], differentiation along mesenchymal phenotypes, distinct surface marker expression [22,23], and specific stem cell features such as self-renewal capacity. Other cell types displaying closely related marker expression like fibroblasts and pericytes complicate discrimination, although these cells represent a more maturated phenotype as compared to MSC. Heterogeneous primary MSC are thus suggested to represent a mixture of different interdependent stroma types [24], together with some subpopulations displaying stem-like characteristics. These enable in vitro culture maintenance for a limited time as compared to constitutively proliferating MSC-like cells representing a cell source with permanently reproducible properties [25,26].

Whereas invasive tumor growth promotes tissue damage, MSC are also recruited to the pro-inflammatory environment of tumors to stimulate repair activities. Thereby, MSC perform a large variety of indirect and direct cell–cell interactions with neighboring tumor-associated cell populations, which confer MSC-mediated tumor-inhibitory and tumor-promoting properties. These opposing MSC functionalities can be displayed simultaneously in the same tumor tissues and strongly depend on local conditions within the tumor microenvironment. In fact, the circumstances are determined by the availability, concentration, and synergy of stimulating factors acting within a small region of the MSC vicinity. Based upon the surroundings in distinct compartments of the tumor tissue, heterogeneous activation of MSC then regulates slowed tumor growth in certain areas while other tumor parts are induced for strong proliferation that is consistent with the often observed inhomogeneous tumor growth [27].

## 3. MSC and Cancer Cell Fusion

### 3.1. Different Hybrid Cancer Cell Lines Following Fusion with MSC

Among others, MSC also support tumor neovascularization and are involved in cancer cell fusion. Cell fusion exhibits chromosomal instabilities and subsequent DNA rearrangements that can create various new recombinations with lethal outcome or acquisition of new properties. Similar to physiological fusion processes, cancer cell fusion also suggests an initial production of tetraploid hybrid cells. Further modification of this unstable situation can result in additional forms of aneuploidy or polyploidy of the cancer hybrid populations and increase tumor heterogeneity. Previous work has listed several established human cancer cell lines that were derived from biopsies of different tissues and tumor entities demonstrating aneuploidy that likely evolved from previous cancer cell fusions [28]. This hypothesis is supported by a model in which tetraploid cells emerge early in carcinogenesis as tumor precursors and develop into aneuploid neoplastic cells with aberrant chromosome numbers [29,30].

Although human MSC display no signs of spontaneous transformation in vitro, a small number of derailed MSC may evolve within a population after autofusion. Cell fusion can also contribute to mesenchymal cell differentiation [31]. Several studies provide evidence that sarcoma tumor types originate from aberrant MSC. Sarcomas can be discriminated in rigid bone tumors and soft tissue tumors whereby Ewing sarcoma represents a mixture of both displaying a poorly differentiated tumor arising in both bone and soft tissues. Sarcomas often represent the tissue type associated with differentiation potential of MSC including osteo-, lipo-, and chondrosarcoma. These sarcomas frequently display chromosome and gene translocations, and several sources indicate an origination from MSC-like populations [32].

**Figure 1:**
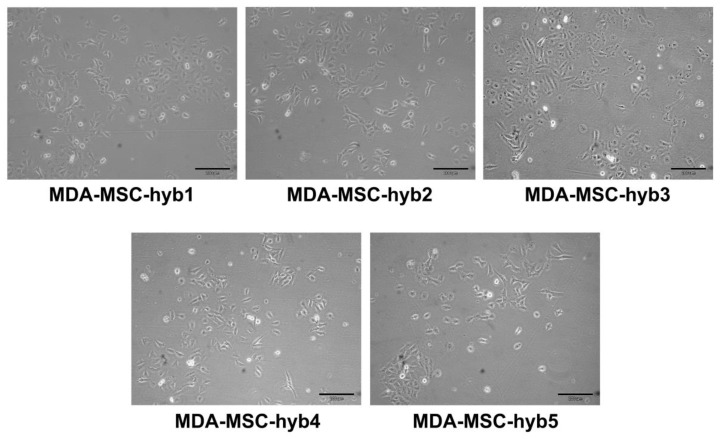
Altered morphologies displayed by different hybrid cancer cells isolated after spontaneous fusion of MDA-MB-231 breast cancer cells with human mesenchymal stroma/stem-like cells (MSC) populations.

During intense cell–cell communication within the tumor stroma, including a mutual exchange of factors, microvesicles/exosomes, and parts of the plasma membrane by trafficking nanotubes or by trogocytosis, MSC eventually can also fuse with various cancer cell types. In particular, breast cancer cell fusion and subsequent generation of breast cancer hybrid/chimeric cells (Figure 1) is observed during close interaction of cancer cells with MSC in the tumor microenvironment [33,34,35]. Accordingly, the generation of new breast cancer hybrid cells is associated with the acquisition of new cellular properties. A variety of different breast cancer hybrid cells could be identified following in vitro fusion of benign neoplastic MCF10A cells or aggressive triple negative MDA-MB-231 breast cancer cells with different individual MSC populations (Table 1). Of interest, the five different populations of MDA-MSC hybrid cells (isolated from spontaneous cell fusions of MDA-MB-231 with different MSC) briefly termed MDA-hyb1 to MDA-hyb5 display distinct morphologies (Figure 1), altered tumorigenic properties, and metastatic behavior.

### 3.2. Enhanced and Reduced Tumorigenicity of Hybrid Cancer Cell Lines after Fusion with MSC

Previous work demonstrated that MDA-hyb1 and MDA-hyb2 cells express different short tandem repeat DNA profiles, pronounced telomerase activities, and increased proliferative capacities, compared to their parental cells. Further characterizations revealed that MDA-hyb2 represent a more MSC-like phenotype than MDA-hyb1. In a xenograft mouse tumor model, these two cancer hybrid cell types also developed a rapidly enhanced tumor growth compared to MDA-MB-231 cells [34]. Although microarray-based mRNA profiling demonstrated a marked up-regulation of genes promoting an epithelial-mesenchymal transition, this was accompanied by significantly elevated formation of distal organ metastases in MDA-hyb2 and even more in MDA-hyb1 cells. For example, MDA-hyb1 cells had developed metastases in lung, liver, spleen, heart, and kidney in most of the animals at a time when metastatic growth in the parental MDA-MB-231 breast cancer cells still remained undetectable and appeared much later. This is in line with previous concepts suggesting cancer cell fusion a potential mechanism for tumor metastases [16]. MDA-hyb1 and MDA-hyb2 cells also demonstrated increased sensitivity to a variety of chemotherapeutic compounds [34]. Other fusion partners of breast cancer cells include tumor-associated macrophages. These fusion processes in vitro and in vivo demonstrated acquisition of macrophage-like properties in the hybrid cancer cells, including expression of the transmembrane adapter protein DAP12 and the macrophage-specific scavenger receptor CD163, which was also detectable in cancer cells of clinical tumor specimen [38,39].

MDA-hyb3 and MDA-hyb4 cells were also isolated by single cell cloning, although after spontaneous fusion of MDA-MB-231 cells with a different MSC population (Table 1). In vitro studies demonstrated a higher proliferative capacity of MDA-hyb3 than MDA-hyb4 cells. When MDA-hyb3 cancer hybrid cells started in vivo tumor development following initial subcutaneous injection by using the mouse xenograft tumor model, however, growth of tumors progressed much more slowly and heterogeneously than among MDA-MB-231 cells. Cancer cell spreading to distal organs was limited during MDA-hyb3-induced tumor growth, with no detectable metastases in lung and kidney, respectively [36]. These findings indicate retarded tumor development, with reduced formation of metastases by MDA-hyb3 cells in comparison to the parental MDA-MB-231 cells. Therefore, in contrast to MDA-hyb1 and MDA-hyb2 breast cancer hybrids, MDA-hyb3 and MDA-hyb4 populations increase tumor heterogeneity by exhibiting reduced tumorigenic properties as compared to their parental counterpart. Although chemotherapeutic responsiveness of MDA-hyb3 and MDA-hyb4 cells and potential cancer stem-like properties still require further examination, these opposite properties of different breast cancer hybrids underscore an altered tumor plasticity after cancer cell fusion of the same breast cancer cells (MDA-MB-231) with different individual MSC populations.

Another MDA-MSC hybrid cell line briefly termed MDA-hyb5 further enlarges the spectrum of tumor plasticity by displaying additional features during tumor development. MDA-hyb5-induced tumors, and distal organ metastases became detectable when tumor growth and metastatic development of the parental MDA-MB-231 cells was already terminated. Whereas neoplastic growth of MDA-MB-231 cells started directly after subcutaneous injection into the mice, a simultaneous transplantation of MDA-hyb5 cells in further mice was associated with initial quiescence of several weeks up to a half year. Following this variable period of cancer cell dormancy, MDA-hyb5 cells started to develop tumors and organ metastases more rapidly, about twice as fast as the parental MDA-MB-231 cells. This suggested a rapid tumor neovascularization and accelerated dissemination of MDA-hyb5 cells after tumor initiation. Comparison of transcripts by RNA microarray analyses revealed an elevated expression of some dormancy-associated genes in MDA-hyb5 versus MDA-MB-231 cells. These differences in gene expression also became apparent when compared to MDA-hyb3 versus MDA-MB-231 and MDA-hyb1 versus MDA-MB-231 cells, respectively. Cell populations without any symptoms can carry dormant cancerous lesions [40]; the majority of these lesions never progress to tumorigenic growth. These in situ tumors are controlled at least in part by the balanced availability of pro-angiogenic growth factors like fibroblast growth factor, vascular endothelial growth factor, platelet-derived growth factor, and IL-8, paralleled by a sufficient supply of statins as inhibitors of tumor neovascularization, including thrombospondin, tumstatin, canstatin, endostatin, and angiostatin. Previous findings demonstrated that accumulation of thrombospondin-1 is involved in breast cancer dormancy [41]. Among others, further dormancy-associated genes include CXCR4 in metastasized breast cancer [42] and TGF-beta2 in prostate cancer [43]. This intermediate state can keep cancer cells dormant in a temporary G_0_′ growth arrest cycle. Cancer cells may require a transient phase of dormancy also for, e.g., adaptation to altered microenvironmental tissue conditions during dissemination and metastases formation, coping with chemotherapeutic drug exposure, or acquisition of new mutations [44]. Multiple coordinated signals, however, may be required to maintain cancer cells in quiescence accompanied by subsequent triggers to escape a transient G_0_′ growth arrest cycle of dormancy and acquire/regain proliferative capacity for tumor development/recurrence.

### 3.3. Potential Cancer Stem Cell-Like Properties in Hybrid Cancer Cell Populations

During MDA-hyb5 tumor explant culture an elevated drug sensitivity was observed. This suggested ongoing functional modifications of these hybrid cancer cells during the rapid tumor development, indicating continuous instability and an increased heterogeneity. Compared to MDA-MB-231 cells, MDA-hyb5 cells displayed different chemotherapeutic sensitivities whereby a detectable unresponsiveness beyond certain concentrations of chemotherapeutic agents indicated a potential resistance within the MDA-hyb5 population. On the other hand, permanent proliferation with self-renewal capacity and enhanced resistance to apoptotic stimuli, including anti-cancer drugs, represent properties of cancer progenitor cells, tumor-initiating cells, and cancer stem cells [45,46]. Spontaneous fusion of certain human breast epithelial with breast cancer cells generates distinct hybrid populations exhibiting cancer stem cell properties [47,48]. Spontaneous fusion of bone marrow-derived MSC with lung cancer cells likewise form new hybrid cancer cell populations displaying altered functions including cancer stem cell-like properties [49]. Normal human mammary epithelial cells as well as patient-derived primary human breast cancer epithelial cells can also spontaneously fuse with MSC occurring within less than 5 min [35]. Other fusion processes with MSC include human SK-OV-3 ovarian cancer cells resulting in the SK-MSC-1 and SK-MSC-2 hybrid cancer cells (Table 1) [37].

Together, these findings suggest spontaneous in vitro cell fusion of MSC with corresponding cancer cells resulting in the formation of various cancer hybrid populations that display completely different properties. Fusion of MSC with breast cancer cells is also detectable in vivo [36,50], which adds to the clinical relevance of cancer hybrid cells in the potential diversification of tumorigenic and metastatic behavior. In vivo cancer cell fusion would influence therapeutic regimens and patient outcome. Nevertheless, evidence for in vivo cancer cell fusion and acquisition of new genomic properties using transplantable cancer cell lines still represents a largely simplified model that limits translational clinical aspects [51].

## 4. Mechanisms of Cancer Cell Fusion

### 4.1. Molecular Signals Involved in Physiological Cell Fusion

Several methods based on cre/lox recombination or using fluorescence gene recombination (e.g., mcherry and green fluorescent protein (GFP)) can be applied to detect cell fusion events. Precise molecular mechanisms for cell fusion processes in general, however, remain to be elucidated. Biophysical requirements include local membrane protrusions, which allow the two joining cell membranes of the cellular fusion partners coming into close proximity. These contacts create microdomains that favor overlapping of the plasma membrane lipid bilayers and cytoplasmic exchanges between the adjacent cells. Some details for cellular hybrid formation have been unraveled in distinct tissues such as placenta. Proteins displaying fusogenic properties as potential ligand for membranous regions are represented by syncytin-1 and -2 predominantly found in syncytiotrophoblasts of placenta tissue. These two proteins evolve from truncated genes originating from viral functions like the HERV-W retroviral envelope genes [52,53] and are also expressed in various solid tumors. In addition, alanine, serine, and cysteine selective transporter-2 ASCT 2 and major facilitator superfamily domain containing 2A (MFSD-2A) can serve as corresponding syncytin adaptor proteins on a neighboring fusion partner cell [52,54]. During trophoblast fusion for development of syncytiotrophoblasts, MFSD-2A has been described as the receptor for syncytin-2 [54,55].

### 4.2. Signaling Events Contributing to Pathophysiological Cell Fusion

In a pathophysiological environment previous findings demonstrated that heterofusion of breast cancer cells with endothelial cells involves endothelial-cell-associated ASCT2, which functions as a receptor for syncytin present on breast cancer cells [56]. In addition, human SK-OV-3 ovarian cancer cells are capable of hybrid cell formation following fusion with human MSC resulting in SK-MSC-hyb1 and SK-MSC-hyb2 hybrid populations. Of interest, the generation of these new ovarian cancer hybrid cells was associated with a less tumorigenic phenotype displaying reduced proliferative capacity in conjunction with a loss of tumorigenic potential [37]. Both fusion partners (SK-OV-3 and MSC, as well as the resulting hybrid populations) exhibited expression of syncytin-2 and MFSD-2A. Although these findings suggested the availability of a fusogenic environment similar to trophoblasts, confirmative data are still missing that would substantiate a clear involvement of syncytins and MFSD-2A in ovarian cancer cell/MSC fusion. This important notice became obvious during breast cancer/MSC fusion. The neoplastic benign human MCF10A breast epithelial cells can undergo cell fusion during co-culture with MSC by simultaneous expression of syncytins, ASCT2, and MFSD-2A, in both fusion partners, likewise indicating fusogenic properties. Down-modulation of each of these fusogenic proteins as well as double knockdown of syncytin-1, ASCT2 and syncytin-2, and MFSD-2A in either of the co-culture populations, however, exhibited no significant differences in hybrid cell formation when compared to nontargeting siRNAs or control co-cultures [35]. These surprising findings demonstrate only minor involvement if any of the known fusogenic factors in the formation of MCF10A-MSC-hybrids, suggesting a different mechanism. Indeed, further examination of potentially associated pathways identified the tumor necrosis factor-alpha (TNF-α) receptor signaling cascade. MCF10A cells produce and release markedly elevated levels of the pro-inflammatory cytokine TNF-α as compared to MSC. Vice versa, TNFR1 and –R2 (TNF receptor-1 and -2) are expressed predominantly in different MSC populations, in contrast to low expression levels of TNFR2 in normal HMEC (human mammary epithelial cells) and in breast cancer cells [35]. Accordingly, MCF10A-mediated TNF-α released contributes to heterofusion by triggering TNF receptors in MSC via TNFR1-associated death domain protein (TRADD) downstream signaling and activation of distinct NF-kB or apoptosis/necroptosis pathways [33,35,57]. Together the findings indicate that different molecular mechanisms favor fusion processes depending on the local environment and the participating cellular fusion partners.

**Figure 2:**
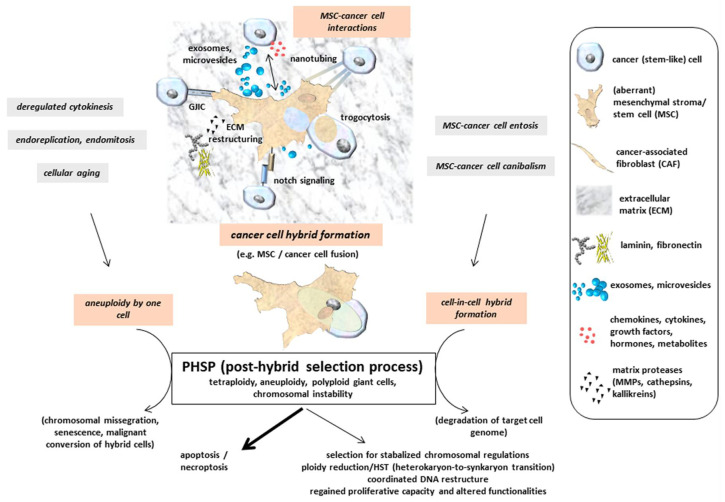
Indirect interactions between MSC and cancer cells are performed by a mutual exchange of a plethora of soluble factors, including microvesicles and exosomes, which may also provide therapeutic vehicles [75,76]. Moreover, direct cell–cell interactions include gap junctional intercellular communication (GIJC), signaling via notch receptor and ligand, and exchange of cytoplasmatic compounds and small organelles (e.g., mitochondria) via nanotubes or trogocytosis. Tight assembly of the two plasma membranes from MSC and cancer cells provide a fusion permissive prerequisite for subsequent hybrid cell formation. Further mechanisms add to the generation of hybrid cells, which undergo a post-hybrid selection process (PHSP) to cope with aneuploidy and chromosomal instabilities. (adapted from [77]).

This hypothesis is further supported by unraveled mechanisms during macrophage fusion or myoblast fusion with a formation of multinucleated giant cells. Signaling molecules involved in the appearance of these fused giant cells include the guanine nucleotide exchange factors Brag2 and Dock180 (dedicator of cytokinesis) [58]. Brag2 converts Arf·GDP to Arf·GTP, and the nucleotide exchange activity is stimulated by phosphatidylinositol 4,5-bisphosphate. Dock180 is a Src-homology 3 protein that interacts with the adaptor protein Crk and activates Rac1 in cooperation with ELMO (engulfment and cell motility) [59]. Rac1 represents a member of the Rho protein family of small GTPases and is involved in the formation of cell-to-cell adhesion, whereby its activity is balanced by the related isoform Rac1b and TGFβ [60]. These signaling complexes promote changes to the actin cytoskeleton that could relay mechanical tension on cell membranes to facilitate fusion and the emergence of multinucleated giant cells by hybrid cell formation [61]. These giant cells or hybrid cell formation followed by aneuploidy and genomic instability could develop senescence or malignant conversion (Figure 2). A small population of giant cells was also observed in a myeloid leukemia cell model during retrodifferentiation from adherent monocyte/macrophage-like cells to non-adherent monoblastoid phenotypes [62,63]. Alterations in the actin and intermediate filament cytoskeletal structures modified by reversible activation of protein kinase C contribute to either cell fusion-mediated formation of giant cells or retrodifferentiation [64,65,66].

## 5. Post-Fusion or Post-Hybrid Selection Processes (PHSPs)

Multinucleated giant cells or fused cancer hybrid cells with tetraploid or aneuploidy sets of chromosomes undergo a post-fusion selection process or more generally a PHSP (post-hybrid selection process) (Figure 2). Appearance of multinucleated giant cells includes the generation of heterokaryons, whereby the parental genomes are located in different nuclei and segregated from one another in contrast to synkaryons. Cell fusion may therefore result in both nuclear forms of hybrids: heterokaryons or synkaryons. When bi- or multinucleated hybrids are generated (heterokaryons), further cell cycle progression by the diverse and uncoordinated nuclear signaling remains questionable. If these hybrid cells with segregated nuclei are capable of cell division, resulting daughter cells can express both parental sets of chromosomes in a single nucleus also called synkaryon [67,68,69]. Synkaryons can be formed directly after interaction and fusion of MSC with epithelial cells, including cancer cells from solid tumors by reorganization of these hybrid cells including nuclear fusion [70]. Due to DNA instabilities in initially formed cancer hybrid cells with synkaryons, chromosomal reduction or reorganization by a PHSP also remains a necessity to enable survival of a genetically stabilized phenotype. Accordingly, fusion-associated MSC hybrid cell formation is accompanied by a recombination of genomic parts from both parental donors in a nuclear hetero-to-synkaryon transition (HST), which can be associated with ploidy reduction during subsequent cell division (Figure 2). Another type of hybrid cells can be formed by engulfment of a target cell, e.g., via cannibalism [71] or entosis-like mechanisms [72] (Figure 2). These forms of cell merger are associated with degradation of the target cell genome. Furthermore, cancer cell fusion tetraploid neoplastic hybrid cells can be generated by derailed cell division mechanisms, including mitotic cleavage failure and cytokinetic imbalances, which represent predominant pathways for tetraploidization in vivo [73]. Aberrant DNA profiles or aneuploidy can also arise during abnormal cell divisions such as endoreplication, endomitosis, or deregulated cytokinesis, as is observed in a variety of different cancer types and derived cancer cell lines [28] (Figure 2). In addition, the proportion of polyploid (including tetraploid) hybrid cells is markedly elevated during the aging process in several tissues [74] (Figure 2).

Hybrid cells represent new populations that require long-term regulatory adaptation since their formation occurs rapidly and in various cases spontaneously. Unsuccessful nuclear reorganization or failed chromosomal restructuring results in termination of a PHSP with subsequent apoptosis/necroptosis (Figure 2). This phenomenon is observed in the vast majority of cancer cell fusion with MSC after single cell selection [34,35,36,37] and most probably also happens to other hybrid cell populations. Accordingly, this substantiates that the successful PHSP-mediated development of viable cancer hybrid cells with proliferative capacity represents a rare event. A PHSP appears to be sensitive to any kind of interference and focused on the cell type. Indeed, previous work reported large differences in the amount of hybrid cell formation, demonstrating an up to 10-fold increase in in vitro fusion of MSC with benign neoplastic MCF10A cells as compared to malignant MDA-MB-231breast cancer cells as fusion partner. In contrast to autofusion of the breast cancer cells, a 10- to 50-fold elevated heterofusion was detectable with MSC, suggesting the involvement of different molecular signals between the fusion partners [35].

Consequently, a PHSP requires a tightly coordinated sequence of selection steps to move the cancer hybrid cells through various unstable but viable intermediate states of chromosomal rearrangement to finally reach a certain level of DNA and chromosomal stability. Mechanisms still need to be elucidated that favorize either syn- or heterokaryon formation and the kind of molecular signals that contribute to nuclear fusion besides cellular fusion. Moreover, orchestration of survival strategies during PHSP remains largely unknown.

One possible pathway of a PHSP includes a ploidy reduction, e.g., by symmetric reduction of tetraploidy to two diploid karyotypes [78]. Alternatively, PHSP can result in chromosomal missegregation and senescence, which may also affect MSC [74,79,80,81] (Figure 2). Potential consequences of cell fusion and hybrid cell formation-mediated polyploidy, aneuploidy, and genomic instability are discussed in a previous model [82] and include a variety of cellular pathways. Therefore, multiple options of cancer cell development after fusion with MSC significantly enhance associated tumor plasticity. This heterogeneity of hybrid cell tumors counteracts therapeutic interventions and worsens patient prognoses.

## 6. Summary

Close plasma membrane approaches and actin/cytoskeletal restructure represent a prerequisite to enable a fusogenic environment between cancer cells and MSC. Distinct signaling pathways in this intermembranous space can relay a rapid but rare cell type-specific fusion event. Nuclear and chromosomal rearrangements are performed stepwise by a PHSP displaying a clonal convergence of the initial fusion populations by elimination, silencing, or stabilizing the surviving new hybrid cancer cells. This reprogramming during a PHSP increases tumor heterogeneity and can also contribute to the generation of cancer stem-like cells, which opens new avenues for further tumor development. Cancer cell fusion with MSC, however, can also reduce tumorigenic properties, as demonstrated by MDA-MSC-hyb3 and –hyb4 breast cancer cells and SK-MSC-hyb1 and -hyb2 ovarian cancer hybrids. Therapeutic approaches therefore require a better understanding of molecular mechanisms underlying a PHSP to predict and potentially regulate the outcome and functionality of hybrid cancer cells. Comparative analysis of less tumorigenic and metastatic hybrid cancer cells with corresponding parental cancer cells further contributes to the identification of vulnerable signaling pathways that may provide therapeutic relevance.

## Figures and Tables

**Table 1:** Different hybrid cancer cell lines following spontaneous fusion with individual hUC-MSC.

Cancer Cell Line Fusion Partner	hUC-MSC Fusion Partner	Hybrid Cancer Cell Line	mRNA Microarray Data	References
MDA-MB-231	MSC051212	MDA-MSC-hyb1	GSE100551	[34]
MDA-MB-231	MSC051212	MDA-MSC-hyb2	GSE100551	[34]
MDA-MB-231	MSC290115	MDA-MSC-hyb3	n.d.	[36]
MDA-MB-231	MSC290115	MDA-MSC-hyb4	n.d.	[36]
MDA-MB-231	MSC030816	MDA-MSC-hyb5	GSE157199	
SK-OV-3	MSC081113	SK-MSC-hyb1	GSE117411	[37]
SK-OV-3	MSC081113	SK-MSC-hyb2	GSE117411	[37]
MCF10A	MSC060616	MCF10A-MSC-hyb1	GSE106756	[35]
MCF10A	MSC060616	MCF10A-MSC-hyb2	GSE106756	[35]

## Abbreviations

ASCT-2	alanine: serine, and cysteine selective transporter-2
GFP	green fluorescent protein
HST	hetero-to-synkaryon transition
hTERT	human telomerase reverse transcriptase
MDA-hyb1 to -hyb5	MDA-MSC-hyb1 to -hyb5 (hybrid cells after fusion of MDA-MB-231 with MSC)
MFSD-2A	major facilitator superfamily domain containing 2A
MSC	mesenchymal stroma/stem-like cells
PHSP	post-hybrid selection process
TNF-α	tumor necrosis factor-alpha
TRADD	TNFR1-associated death domain protein
